# DNA-Based Enzyme Reactors and Systems

**DOI:** 10.3390/nano6080139

**Published:** 2016-07-27

**Authors:** Veikko Linko, Sami Nummelin, Laura Aarnos, Kosti Tapio, J. Jussi Toppari, Mauri A. Kostiainen

**Affiliations:** 1 Biohybrid Materials, Department of Biotechnology and Chemical Technology, Aalto University, P.O. Box 16100, Aalto 00076, Finland; sami.nummelin@aalto.fi (S.N.); laura.aarnos@aalto.fi (L.A.); 2 Department of Physics, University of Jyvaskyla, Nanoscience Center, P.O. Box 35, Jyväskylä 40014, Finland; kosti.t.o.tapio@jyu.fi (K.T.); j.jussi.toppari@jyu.fi (J.J.T.)

**Keywords:** DNA nanotechnology, DNA origami, self-assembly, enzyme, cascade reactions, DNA nanodevice, DNA sensors, drug-delivery, nanomedicine

## Abstract

During recent years, the possibility to create custom biocompatible nanoshapes using DNA as a building material has rapidly emerged. Further, these rationally designed DNA structures could be exploited in positioning pivotal molecules, such as enzymes, with nanometer-level precision. This feature could be used in the fabrication of artificial biochemical machinery that is able to mimic the complex reactions found in living cells. Currently, DNA-enzyme hybrids can be used to control (multi-enzyme) cascade reactions and to regulate the enzyme functions and the reaction pathways. Moreover, sophisticated DNA structures can be utilized in encapsulating active enzymes and delivering the molecular cargo into cells. In this review, we focus on the latest enzyme systems based on novel DNA nanostructures: enzyme reactors, regulatory devices and carriers that can find uses in various biotechnological and nanomedical applications.

## 1. Introduction

In order to maintain complex metabolic pathways, nature uses compartmentalization and spatial organization of metabolically active units to separate specialized functions, control activity and gain specificity. In the cell, specific organelles control the location and crowding of enzymes, which has a profound effect on their spatial action, and ultimately allows different metabolic pathways to operate at the same time in close proximity, but in different compartments. For example, electron transport and oxidative phosphorylation are handled by the mitochondrion, whereas at the same time glycolysis and fatty acid biosynthesis take place in the cytosol. Furthermore, multiple enzymes responsible for the individual reaction steps in a metabolic pathway are often combined into a single multifunctional enzyme or complex in order to enhance and control the reaction cascade or cycle. Fatty acid synthase is one of the prime examples: in animals it combines two identical protein chains that contain seven different catalytic activities required for the biosynthesis of fatty acids. 

Although highly desirable, the programming of chosen reaction cascades and creating artificial systems that can position and confine different enzymes is still far from the complexity achieved by nature. Controlling chemical reactions by using self-assembled nanoscale reactors has consequently emerged as an active area of research [1]. Simple compartmentalization of enzymes has already been achieved by using different nanoscale reactors. Examples of such systems include sol-gel materials [2], polymersomes [3], protein cages [4,5,6] and crystalline structures [7,8,9]. Porous polymersomes were used as nanoreactors to anchor three different enzymes into separate locations: the lumen, bilayer membrane and surface. Protein cages, such as virus-like particles, have been utilized to pack different enzymes that perform a coupled cascade reaction densely inside a porous protein shell. Finally, for example, metal-organic frameworks have been designed to trap and encapsulate enzymes and shown to prevent their aggregation and denaturation. All of the above-mentioned systems are prime examples on how the positioning, separation and clustering of enzymes can be controlled.

**Figure 1:**
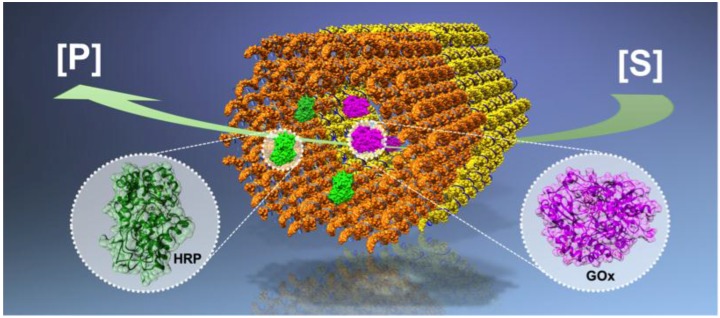
A schematic view of an enzymatic nanoreactor built from DNA ([S] = substrate, [P] = product). By taking advantage of the high addressability and modularity of the DNA nanostructures, enzymes can be attached and arranged with nanometer-scale precision. As an example, glucose oxidase (GOx, purple)–horseradish peroxidase (HRP, green) cascade pairs have been assembled into a confined reaction space provided by two tubular DNA origami nanostructures (orange and yellow cages).

In this review, we focus on complexes and systems that involve functional enzymes and novel DNA nanostructures (an example of such a system is depicted in Figure 1). These sophisticated DNA nanostructures can be programmed to form precise and controllable arrangements of enzymes at the nanoscale, and these systems are particularly engaging for various applications in bioengineering and nanomedicine. We have divided this review into four main sections. First, we briefly summarize the development in structural DNA nanotechnology and discuss how DNA motifs can be combined with functional enzymes (Section 2). Section 3 is devoted to (static) enzymatic nanoreactors, and Section 4 covers the enzymatic regulatory devices with mechanical function. Finally, in Section 5, containers and carriers for protecting and delivering enzymes are discussed.

## 2. Building with DNA Molecules and Enzymes

### 2.1. DNA Nanostructures

**Figure 2:**
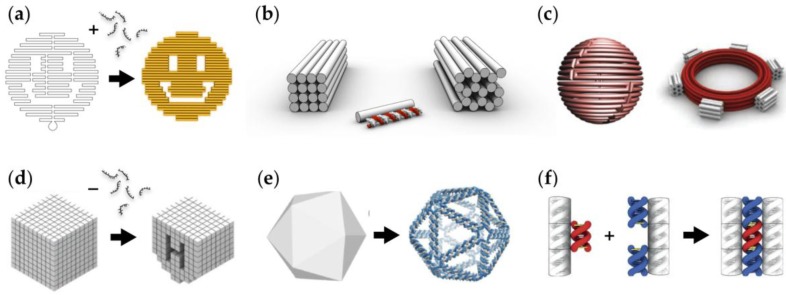
(**a**) A DNA origami technique. A long scaffold strand is folded into a desired shape with the help of short staple strands [14]; (**b**) Multilayer DNA origami in square and honeycomb lattice [15,16]; (**c**) DNA origami with curvatures and bends [17,18]; (**d**) Scaffold-free fabrication of DNA nanoshapes. Numerous target shapes can be fabricated by selecting subsets of strands from the cubic-like ‘molecular canvas’ [19]; (**e**) A fully automated top-down design method to create meshed DNA origami structures [21]; (**f**) DNA origami structures can be glued together by taking advantage of the blunt-end stacking and the shape-complementarity of the origami units [22]. (**a**) is reproduced with permission from [14]. Copyright Nature Publishing Group, 2006. (**b**) is reproduced with permission from [16]. Copyright Nature Publishing Group, 2011. A sphere in (**c**) is reproduced with permission from [17]. Copyright The American Association for the Advancement of Science, 2011. A gear-like object in (**c**) is reproduced with permission from [18]. Copyright The American Association for the Advancement of Science, 2009. (**d**) is reproduced with permission from [19]. Copyright The American Association for the Advancement of Science, 2012. (**e**) is reproduced with permission from [21]. Copyright The American Association for the Advancement of Science, 2016. (**f**) is reproduced with permission from [22]. Copyright The American Association for the Advancement of Science, 2015.

Nadrian ‘Ned’ Seeman postulated around 30 years ago that deoxyribose (DNA) molecules could be used as building material in creating complex predesigned nanostructures through molecular self-assembly [10]; sequence-complementary parts of single-stranded DNA (ssDNA) molecules can be hybridized into double-stranded DNA (dsDNA) domains (via Watson-Crick base-pairing) and therefore into larger programmed shapes. Since then, structural DNA nanotechnology has enjoyed a rapid progress; numerous complex nanostructures and different fabrication techniques have been introduced [11] (Figure 2). A great deal of the first compelling DNA assemblies were based on tile-like structures that enabled fabrication of two-dimensional (2D) [12] and three-dimensional (3D) crystals [13], but nevertheless, the huge upturn in the field was the invention of the ‘DNA origami’ technique [14] (Figure 2a). The origami approach is based on folding a long single-stranded DNA scaffold strand into a desired shape with the help of a set of short oligonucleotides (staples), and it has now become a widely accessible and exploited method to fabricate custom, modular and spatially-well-defined 2D [14] and 3D nanostructures with complex curvatures, bends and twists [15,16,17,18] (see Figure 2b,c). Later on, methods based on scaffold-free fabrication [19] (Figure 2d), polyhedral rendering [20,21] (Figure 2e) and shape-complementarity [22] (Figure 2f) were introduced.

In general, the DNA-based assembly of nanostructures is a highly parallel technique, and the nanometer-scale addressability of the created objects makes it an intriguing approach for developing novel bionanotechnological applications [11]. To date, loads of implementations based on DNA nanostructures have been presented, such as tunable plasmonic devices and metallic nanoshapes [23,24], rulers for optical imaging [25], structures for nanoelectronics [26,27], artificial ion channels for transporting or sequencing molecules [28], and nanorobots for targeted drug delivery [29]. Moreover, as discussed in this review, DNA nanostructures provide an excellent foundation for designing enzymatic reactors and complex catalytic systems at the nanoscale.

### 2.2. DNA-Enzyme Conjugates and Arrays

As discussed above, DNA structures can be used as templates for various molecules, inorganic nanoparticles, and equally for functional enzymes [30]. Enzymes can be conjugated directly to an oligonucleotide (part of a DNA structure) or they can be attached to DNA through a specific binding motif [30]. In general, it is important that the enzyme activity is retained in the conjugation; a chosen enzyme should not be modified chemically or genetically [31]. For example, sequence-specific DNA-binding proteins can be used as adaptors in attachment [32], and their use can help to maintain the enzyme activity in the conjugation.

To date, there exist numerous reports of utilizing simple nucleic acid motifs to assemble functional enzymes and to organize chemical reactions with programmability [33,34,35,36]. In addition, it has been shown that by utilizing DNA-based self-assembly, structurally-well-defined protein arrays [30,37] and DNA-enzyme crystals [7] can be created. Along these lines, this review discusses recent progress in creating smart enzyme reactors, dynamic regulators, protein containers and carriers by taking advantage of state-of-the-art DNA nanostructures, such as DNA origami.

## 3. Enzyme Reactors and Cascades

An enzyme reactor typically contains one or more enzymes, which catalyze a desired reaction. The purpose of the enzyme reactor is usually to maximize the reaction efficiency via compartmentalization or by bringing the reaction counterparts in close proximity to each other. By utilizing designed DNA nanostructures with high addressability, enzymes can be attached to them with nanoscale precision. This is a key factor for enzyme functions; a substrate can only bind to an enzyme in a specific orientation, and on the other hand, the proximity of the compounds provided by the DNA templates could significantly enhance the enzymatic reaction rates [38,39]. In addition, it is essential to control the channeling of the substrate and the reaction intermediates of the enzyme cascades [40]. In many cases, compartmentalization could be used to efficiently separate and arrange simultaneous reactions and reaction compounds similar to complex natural systems [41]. Moreover, enzyme reactors can be equally utilized to study enzyme functions and reaction pathways [42]. In this section, recent examples of using DNA nanostructures to build (static) nanoreactors for biosensing and molecular-scale diagnostics are discussed (see also Table 1). 

**Figure 3:**
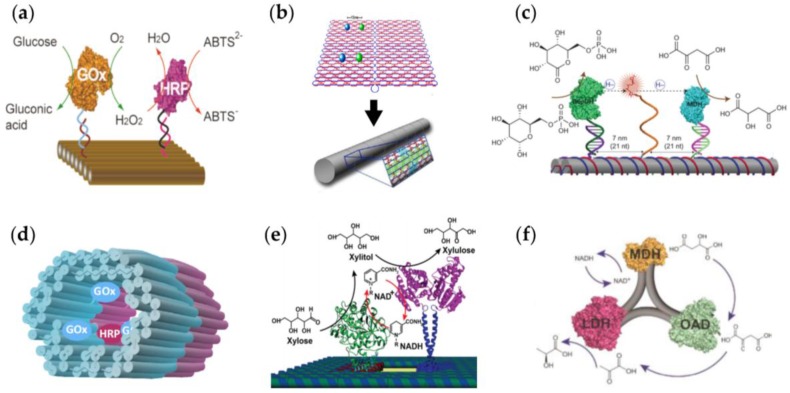
(**a**) A glucose oxidase (GOx) – horseradish peroxidase (HRP) enzyme cascade pair assembled on a rectangular DNA origami [43]; (**b**) A rectangular DNA origami shape with attached enzyme cascade pairs (GOx and HRP) can be rolled into tubular shapes [44]; (**c**) A swinging arm for cofactor transfer between the enzymes (malate dehydrogenase (MDH) and glucose-6-phosphate dehydrogenase (G6pDH)) assembled on a DNA tile [45]; (**d**) A modular and tubular DNA origami-based enzyme cascade (GOx and HRP) nanoreactor [46]; (**e**) An artifical enzyme cascade (xylose reductase (XR) and xylitol dehydrogenase (XDR)) performing a cofactor coupled cascade reaction on DNA origami [47]; (**f**) An artificial three-enzyme (lactate dehydrogenase (LDH), MDH and oxaloacetate decarboxylase (OAD)) pathway organized using a DNA nanostructure [48]. (**a**) is reproduced with permission from [43]. Copyright American Chemical Society, 2012. (**b**) is reproduced with permission from [44]. Copyright American Chemical Society, 2013. (**c**) is reproduced with permission from [45]. Copyright Nature Publishing Group, 2014. (**d**) is reproduced with permission from [46]. Published by The Royal Society of Chemistry, 2015. (**e**) is reproduced with permission from [47]. Copyright American Chemical Society, 2016. (**f**) is reproduced with permission from [48]. Copyright John Wiley and Sons, 2016.

In the enzyme cascade system presented in Figure 3a, glucose oxidase (GOx) catalyzes the oxidization of glucose (substrate) in the presence of oxygen to generate gluconic acid and a hydrogen peroxide (H_2_O_2_) intermediate, which, in turn, serves as a substrate for horseradish peroxidase (HRP) (HRP reduces H_2_O_2_ into water). Simultaneously, the presence of H_2_O_2_ results in the protonation of the ABTS^2−^ (2,2′-azinobis-(3-ethylbenzthiazoline-6-sulfonate) dianion, and hence, an ABTS^−^ radical anion is generated (ABTS^−^ acts as a reporter of the enzyme activity). The diffusion distance of the hydrogen peroxide limits the rate of this enzyme cascade reaction since HRP has a much higher turnover rate than GOx. Fu et al. studied interenzyme substrate diffusion by using a rectangular DNA origami tile as a platform to preorganize GOx-HRP pairs in a distance-dependent manner [43]. The highest cascade activity was obtained when the interenzyme distance was 10 nm. Importantly, the activity was about 15 times higher than the control sample that contained unbound enzymes. A drastic decrease in activity was observed as the interenzyme distance was adjusted to 20 nm, and the activity was further decreased gradually as the distance was increased up to 65 nm.

Inspired by the above-mentioned work, Fu et al. [44] designed rectangular (100 nm × 70 nm) DNA tiles with GOx-HRP cascade pairs precisely positioned 15 nm apart from each other. By using sticky-end extensions on the top and bottom edges of the DNA origami rectangles, they induced the tile to form short DNA nanotubes (Figure 3b). Efficiency of the enzyme cascade reaction was quantitatively measured using an excess amount of reactant glucose and the chromogenic reaction of the reporter ABTS^2−^ (substrate for HRP). The activity was highest when the enzymes were located in a confined nanospace within the DNA nanotube. When the enzymes were attached to the semiconfined planar DNA tile, the activity was lower, but still higher than that of free cascade controls, which showed the lowest activity. Hence, these nanoscale bioreactors provide access to an artificial system for studying biological processes in organized cell-mimicking environments.

Swinging arms are key constituents of sequenced catalytic transformations in many naturally occurring multi-enzyme complexes. The arm is commonly a chemical group covalently attached to the enzyme complex via a flexible linker that enables the direct transfer of substrate molecules between multiple active sites within the complex. Fu et al. [45] constructed a DNA nanostructure for assembling a multi-enzyme system that is equipped with an artificial swinging arm. The arm was designed to efficiently channel hydride transfer between two dehydrogenases. The whole design is illustrated in Figure 3c. The nanostructure complex utilized a two-enzyme cascade composed of glucose-6-phosphate dehydrogenase (G6pDH) and malic dehydrogenase (MDH) positioned on a DNA double-crossover (DX) tile scaffold. In the cascade sequence G6pDH catalyzes the oxidation of glucose-6-phosphate and the reduction of NAD^+^ (nicotinamide adenine dinucleotide, oxidized) to NADH (nicotinamide adenine dinucleotide, reduced). In the second cycle, MDH catalyzes the reduction of oxaloacetate to malic acid using the NADH produced by G6pDH. The swinging arm, an NAD^+^-equipped poly-thymine (poly-T) oligonucleotide (20 nucleotides long), was adhered to the DNA tile surface exactly halfway between the anchored enzymes G6pDH and MDH. The swinging arm’s capability to boost dehydrogenase activity in complexes containing one enzyme coupled to a single NAD^+^ arm was measured individually in bulk solution for three distances (7, 14 and 21 nm). The highest activity for both G6pDH and MDH was observed at the 7 nm distance showing ca. 25-fold enhancement of activity compared to an enzyme system in the presence of the same concentration (100 nM) of freely diffusing NAD^+^. In the same experimental conditions, the activity of the fully assembled G6pDH–NAD^+^–MDH two-enzyme nanostructure with a swinging arm is ca. 90-fold higher than that obtained using the same two-enzyme complex but with freely diffusing NAD^+^.

Linko et al. [46] designed and fabricated an enzyme reactor, which consists of two distinct tubular 3D DNA origami building blocks with either GOx or HRP enzymes anchored inside the origami compartment through biotin–NeutrAvidin (NTV) binding (Figure 3d). Both units were fabricated separately, and ‘glued’ together via a programmable DNA base-pairing by hybridizing 32 short (three to six bases) sequences. The short sequences that were sticking out at the end of one unit were paired with free scaffold sites located at the edge of another unit. The other end of the origami unit was passivated by overhanging single-stranded poly-T sequences (8 nucleotides) in order to prevent the formation of multimers. The catalytic activity of a two-unit nanoreactor was monitored in the environment containing excess amounts of d-glucose as a reactant and 3,3′,5,5′-tetramethylbenzidine (TMB) as a reporter in order to achieve a reaction that is restricted by the diffusion rate of the intermediate product H_2_O_2_. Compared to the control samples (similarly prepared samples but without NTV binding sites for enzymes), the assembled twin-unit nanoreactor has much higher activity, thus indicating that unspecific binding between enzymes and origami structures is insignificant.

Ngo et al. [47] introduced cofactor-coupled cascade reactions on a DNA origami scaffold. The cascade was based on the d-xylose metabolic pathway, and combined two enzymes: xylose reductase (XR) and xylitol dehydrogenase (XDR). The enzymes were attached to the DNA scaffold with DNA-binding protein adaptors, the zinc finger protein (zif268) and the basic leucine-zipper protein (GCN4). The cascade mechanism relies on the recycling of cofactor NADH between the enzymes, which is possible due to their close proximity. Within the metabolic pathway of xylose, the first enzyme XR converts xylose into xylitol by consuming the cofactor NADH. The produced xylitol and NAD^+^ are both simultaneously transported to the second enzyme XDH, which converts xylitol into xylulose by consuming NAD^+^ to recycle the NADH cofactor (Figure 3e).

Liu et al. [48] assembled an artificial three-enzyme pathway on a series of DNA nanoscaffolds in order to study the dependence of their activities. They measured the activities of an MDH-OAD-LDH (malate dehydrogenase–oxaloacetate decarboxylase–lactate dehydrogenase) cascade with variable spatial distances and geometric arrangements. The three-enzyme pathway (Figure 3f) starts with the MDH-catalyzed oxidation of malic acid to oxaloacetate (OAA) and the simultaneous reduction of NAD^+^ to NADH. In the next cycle OAD converts OAA into pyruvic acid and inhibits its conversion back to malic acid. In the third cycle LDH consumes the reduced NADH and pyruvic acid to produce lactic acid. Unlike the above-mentioned two-enzyme systems, the overall activity of the three-enzyme pathway was more dependent on the geometric patterns that arranged enzymes within a short distance (10–30 nm) of each other rather than with interenzyme spacings. By optimizing the geometric patterns of the three enzymes, a five-fold activity enhancement was obtained compared to the unassembled free enzymes. In addition, the depletion of the pathway intermediates was very efficient in the assembled enzyme systems with little detectable NADH in the bulk solution, indicating that nearly all NADH was coupled into the enzyme pathway without leakage.

## 4. Enzymatic Nanodevices with Motion

Besides the static nanoreactors discussed in the previous section, there are compelling examples of in vitro nanodevices that can control enzyme activity. These devices can be switched between an active and inactive state by introducing a specific trigger. The triggers are usually DNA strands that are able to perform preprogrammed strand displacement reactions. Alternatively, some of the systems can autonomously regulate the reaction(s). Here, a few examples of mechanical regulatory DNA-enzyme devices, autonomous molecular systems and their working principles are reviewed (see also Table 2).

### 4.1. Mechanical Regulatory DNA-Enzyme Devices

**Figure 4:**
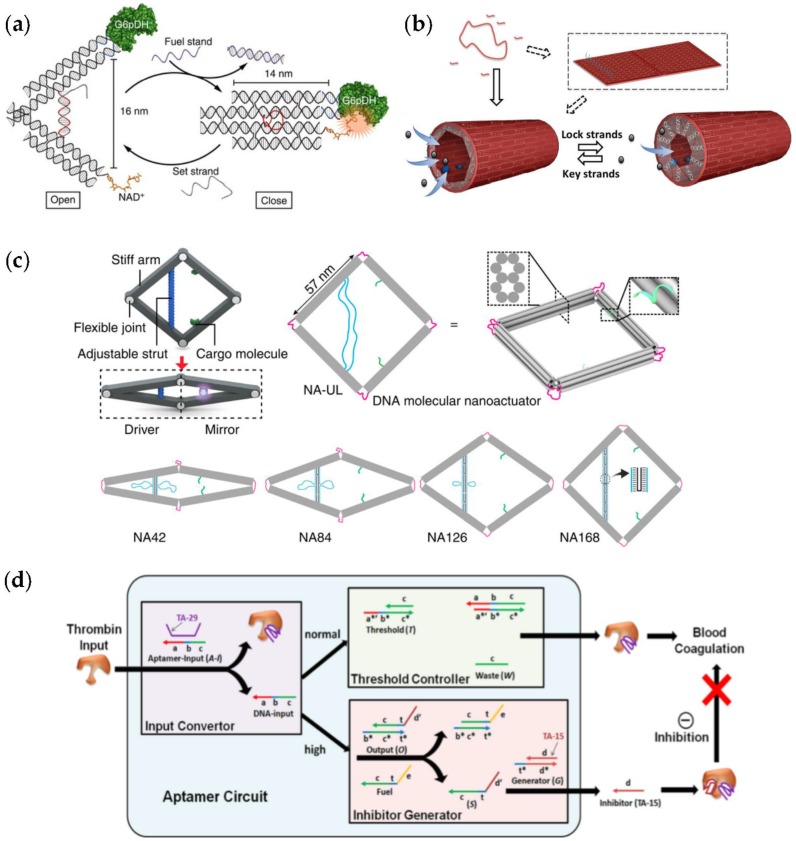
(**a**) Nanotweezers to regulate enzyme activity [49]; (**b**) Tubular nanoreactor with switchable lid to control the flowthrough of the reaction compounds [53]; (**c**) DNA origami nanoactuator that can be driven by, e.g., single-stranded DNA (ssDNA) strands or restriction enzymes [54]; (**d**) Aptamer-based logical molecular circuit to control thrombin activity [55]. (**a**) is reproduced with permission from [49] Copyright Nature Publishing Group, 2013. (**b**) is reproduced with permission from [53]. Copyright The Royal Society of Chemistry, 2016. (**c**) is reproduced with permission from [54]. Published by Nature Publishing Group, 2016. (**d**) is reproduced with permission from [55]. Copyright American Chemical Society, 2012.

Liu et al. [49] employed a DNA tweezer nanostructure to actuate the reaction between a G6pDH/NAD^+^ enzyme-cofactor pair. In this construct (Figure 4a), the enzyme and cofactor were attached to two different ca. 14-nm-long arms. Actuation of the enzyme function was achieved by switching between open and closed states of the tweezers, in other words by spatially separating the enzyme-cofactor pair for inhibition or bringing the pair together for activation, respectively. In the reaction cycle, NAD^+^ is first reduced to NADH by G6pDH. Then, phenazine methosulfate (PMS) catalyzes electron transfer from NADH to resazurin, which produces strongly fluorescent resorufin. In the tweezer geometry, a 25-nucleotide (nt) ssDNA oligomer connected the ends of the tweezer arms and served as a structural regulatory element to control the state of the system. The open state can be attained by disrupting the hairpin via hybridization between a complementary set strand and a hairpin, thus generating a rigid ca. 16-nm-long dsDNA domain between the ends of the tweezer arms. By adding a fuel strand (fully complementary to the set strand) to the system, a hairpin is released by a strand-displacement mechanism and the tweezers are switched back to the closed state. Opening and closing mechanisms have been further optimized by Dhakal et al. [50].

Moreover, Xin et al. [51] used similar nanotweezers and chose the GOx-HRP cascade as a model to demonstrate the reversible regulation of the enzyme cascade reaction. The DNA machine was comprised of double-crossover (DX) motifs, which formed two rigid arms (glued together by an immobile four-way junction). A DNA motor, which can switch between a stem-loop and a double-helix structure driven by a strand displacement reaction, was incorporated into the middle of the DNA machine to cycle between open and closed states. This kind of device could also be used to reversibly regulate the target binding affinity of a thrombin protein, as shown by Chou et al. [52].

Wang et al. [53] prepared a DNA origami nanochannel as a scaffold for monitoring the GOx-HRP cascade reaction. The channel, 100 nm in length and 22 nm in diameter, was formed by rolling up a rectangular origami object with the help of sticky ends, placed as extensions at the top and bottom helices of the sheet-like structure (depicted in Figure 4b). A row of 11 staple strands, called shutter strands, which contain 15 nucleotides long overhangs in an upright position to the concave side, formed a shutter at the end of the nanochannel which can control the opening and closing of the channel upon stimuli. By adding the ‘lock strands’, i.e., ssDNA molecules complementary to the 15 nt overhangs, rigid DNA duplexes were formed, resulting in an efficient closing of the shutter. Reopening the nanochannel was achieved by using fully complementary ‘key strands’. The key strands hybridized with the lock strands, displacing them from the channel and therefore opening the shutter. Reversibility of the shutter mechanism was demonstrated with the sequential addition of 23 nt lock and key strands, performing a cycle change: open-close-open. The cycle was monitored with UV-vis spectroscopy; when the shutter was closed, the efficiency of the cascade reaction in the nanochannel dropped, indicating that the flow of substrates was efficiently obstructed.

Ke et al. [54] developed a nanoactuator (shown in Figure 4c) that consists of four stiff, 10-helix-bundle arms, assembled into a rhombus shape. The actuator is equipped with a mechanical linkage in each corner to enable movement of the one half (the driver, left side) to be expressed at the other half (the mirror, right side). The arms in the design are connected with two single-stranded scaffold segments (light blue), and the strut length (left side) can be adjusted by hybridizing the segments with ‘strut-locking’ strands of predetermined lengths (lower panel in Figure 3c). Both arms on the right side contain short ssDNA extensions that enable immobilization of cargo molecules (green strands). By attaching split enhanced green fluorescent protein (eGFP) to the actuator, the authors constructed a DNA-protein hybrid nanostructure, which showed tunable fluorescent behaviors via long-range allosteric regulation (the binding of an effector molecule controls the global shape of the protein). Moreover, the nanoactuator could find uses as a stimuli-specific sensor. The stimuli can be a change in the buffer compounds or for example the introduction of restriction enzymes (such as restriction endonuclease BamHI).

### 4.2. Autonomous Molecular Systems

DNA nanostructures can be used to control enzymes that are free in the environment. Autonomous molecular systems are able to sense the environment, process the information by a predefined signal-processing algorithm, and finally act based on the results of the computation. Using autonomous molecular systems, the concentration and/or activity of enzymes could be controlled in cells or extra-cellular liquids. These artificial biochemical circuits may enable boolean processing and programmability, and they could also be exploited to monitor chemical reactions and to regulate gene expressions. Briefly, it is possible to perform logic gate operations, amplify and restore signals, create cascades and construct feedback loops. This is all based on programmable nucleic acid base-pairing interactions. Aptamers (special single-stranded oligonucleotides), based on their secondary and tertiary structures, have the potential to specifically interact with enzymes, other proteins and molecules as well as with viruses and cells. Aptamers can also regulate enzyme activity and other protein functions. 

As an example of an autonomous regulator, we describe a logical molecular circuit to control α-thrombin activity. The circuit by Han et al. [55] and its working principle are depicted in Figure 4d. The enzyme α-thrombin is a protease that aids blood coagulation by converting fibrinogen to fibrin.

The circuit is based on ssDNA strand displacement reactions and includes three modules: (a) the input convertor that converts the thrombin input into a DNA sequence that is used later in the cascade reactions of the system; (b) the threshold controller that sets the threshold concentration to preserve the regular throbmin activity level; and (c) the inhibitor generator that suppresses excessively high thrombin activity once the activity surpasses the threshold value. The full circuit is depicted in Figure 4d. The input convertor is a duplex consisting of a TA-29 aptamer and an ssDNA molecule (Aptamer-Input in Figure 4d). A thrombin molecule can bind to the TA-29 and therefore displace the ssDNA. In this way, the thrombin input is converted to an ssDNA input that could be read by the later modules of the circuit. When the ssDNA input enters the threshold controller, it results in an inert ssDNA molecule (Waste in Figure 4d) through a toehold-exchange reaction of the ssDNA input and the threshold duplex (Threshold in Figure 4d). If the concentration of the ssDNA input is higher than the predetermined threshold concentration, the excess ssDNA input enters the inhibitor generator, which is also based on toehold-exchange reactions. The order of these two reactions/modules is determined by the differences in the thermodynamic stability and the reaction kinetics of the DNA strands. In this case, a longer toehold makes the threshold reaction more favorable.

The inhibitor generator module generates an ssDNA output (S in Figure 4d) as the ssDNA input displaces it from an output duplex (Output in Figure 4d). The signal is amplified with the catalytic help of fuel strands (Fuel in Figure 4d). The fuel strand displaces the ssDNA input from the duplex, thereby releasing the input to react with another output duplex. Thus, a single input can produce multiple outputs. Finally, the amplified ssDNA output (S) reacts with a duplex that involves a TA-15 aptamer (Generator in Figure 4d). TA-15 is released, and subsequently it binds to the fibrinogen exosite of the thrombin molecule, inhibiting its activity. Overall, this system serves as a compelling example of an autonomous DNA-based enzyme system, but the sustainability of the system still remains a challenge. The system was not able to recycle the DNA-based cascade components and the modules needed constant administration.

## 5. Enzyme Containers and Carriers

As seen above, DNA nanostructures could perform preprogrammed tasks and efficiently react to triggering signals. These features could be extremely useful in nanomedical applications, such as delivering enzymes into cells (e.g., in enzyme replacement therapy). In general, delivering enzymes into cells can be rather challenging since the transport efficiency is charge- and size-dependent. In addition, one of the concerns is that the stability of enzymes may be easily compromised, which could lead to malfunctioning. However, by using designed and biocompatible DNA nanostructures as carriers, the cargo could be protected and released in a controllable way. In this section we discuss the desired properties of DNA containers and review potential DNA nanostructures and protection mechanisms that could find uses in efficient intracellular delivery of enzymes (see also Table 3).

### 5.1. DNA Containers for Enzymes

**Figure 5:**
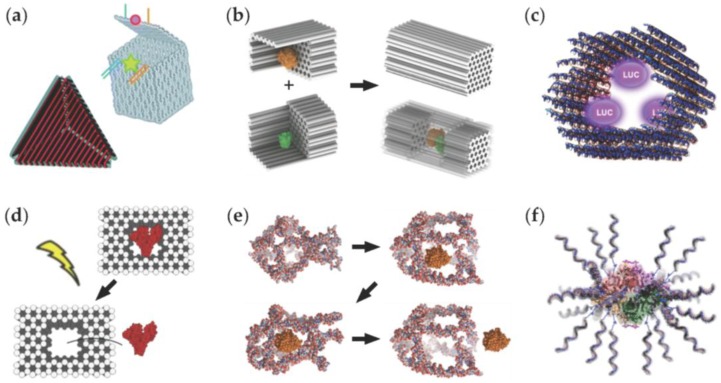
(**a**) Hollow DNA origami containers: a box with a switchable lid [56] and a tetrahedron [57]; (**b**) Two half-cages equipped with glucose oxidase (GOx, orange) and horseradish peroxidase (HRP, green) can form a closed box and encapsulate the enzymes. The closed box efficiently protects the assembled enzyme cascade pair from protease digestion [59]; (**c**) A DNA origami-based nanocarrier loaded with luciferase (LUC) enzymes. The enzyme activity can be tuned by coating the carrier with cationic polymers [60]; (**d**) A light-triggered release of proteins such as bovine serum albumin (BSA) from DNA origami container [61]; (**e**) A DNA cage that can trap and release an enzyme (HRP) through the temperature-controlled conformational changes [62]; (**f**) β-galactosidase (β-gal) can be coated by DNA strands for significantly enhanced cellular delivery [63]. A box with a lid in (**a**) is reproduced with permission from [56]. Copyright Nature Publishing Group, 2009. A tetrahedron in (**a**) is reproduced with permission from [57]. Copyright American Chemical Society, 2009. (**b**) is reproduced with permission from [59]. Published by Nature Publishing Group, 2016. (**c**) is reproduced with permission from [60]. Copyright The Royal Society of Chemistry, 2016. (**d**) is reproduced with permission from [61]. Copyright American Chemical Society, 2016. (**e**) is reproduced with permission from [62]. Copyright American Chemical Society, 2013. (**f**) is reproduced with permission from [63]. Copyright American Chemical Society, 2015.

Andersen et al. were the first to generalize the DNA origami technique to 3D objects [56]. They created a DNA box (42 × 36 × 36 nm^3^ in size) that can be opened by introducing the specific DNA ‘keys’. Six DNA origami sheets were folded along the single-stranded M13 scaffold and further assembled into a 3D box with a lid (Figure 5a, right). The lid was functionalized with a dual lock-key system comprised of double-helices with single-stranded extensions. The attachment of two fluorophores, Cy3 and Cy5, to the adjacent faces of the box enabled the detection of the lid opening and closing by fluorescence resonance energy transfer (FRET).

Ke et al. [57] presented a 3D molecular cage with tetrahedron geometry (estimated inside volume of 1.5 × 10^−23^ m^3^ closed by triangular faces). The DNA tetrahedron was assembled in a single-step process where the scaffold DNA, forming four planar adjacent triangular faces, was mixed together with ssDNA staple strands and slowly annealed to form a desired shape. Figure 5a (left) shows a 3D schematic drawing of the scaffold arrangement in the DNA tetrahedron with seams eliminated. Moreover, Kuzuya and Komiyama [58] fabricated a box-shaped 3D DNA origami by folding the scaffold strand into an open complex of two tetrahedral motifs and then selectively closed the box by adding the appropriate staple strands. The size of the resulting 3D origami (45 × 42 × 35 nm^3^) is quite similar to that of the virus capsids. In general, these DNA containers could enable the selective capture of various guest molecules inside the motif, and they might be suitable for, e.g., harboring enzymes. DNA containers could efficiently protect the enzymes from biological degradation through proteases while maintaining their activities since small substrates can access the container through the small openings in the DNA mesh.

Zhao et al. [59] showed how an active GOx-HRP enzyme cascade pair can be encapsulated and protected from protease digestion using a designer DNA cage. The enzymes were attached to DNA origami–based half-cages (Figure 5b) and the complementary halves were subsequently combined into a cubic DNA box/cage. The walls of the design contain nanopore-like channels that allow the diffusion of small molecules, such as substrates and products, but block the proteases. The cage was tested with six different metabolic enzymes and, as the result, five of the six tested enzymes exhibited turnover numbers four- to ten-fold higher than that of the free enzyme.

Kiviaho et al. [60] fabricated a hexagonal and tubular DNA origami carrier, which was equipped with streptavidin-Lucia enzymes via biotinylated binding sites (Figure 5c). Enzyme-loaded DNA origami was coated with three different cationic polymers (PDMAEMA, PDMAEMA-PEG and PDMAEMA-PEG-PDMAEMA) through electrostatic interactions. The authors studied the effect of different polymer coatings on the luminescence decay rates of luciferase (LUC) enzymes (attached to DNA origami). LUC enzymes showed appropriate catalytic activity in all samples with different polymer concentrations (0, 10, 100 and 1000 polymers per DNA origami), indicating that the added polymers are not capable of blocking the enzyme activity completely. By adjusting the amount of polymer in the coating, it was possible to tune the characteristic enzymatic reaction rates of the enzymes. Moreover, it was shown that the polymer-origami complexes and the polymers themselves were not significantly toxic to human epithelial cells.

Kohman et al. [61] introduced a method to encapsulate bioactive molecules such as streptavidin and bovine serum albumin (BSA) within DNA nanostructures and release them using short (<60 s) pulses of light (Figure 5d). The strategy involved organic photolabile linker, which was reacted with cargo molecules and subsequently conjugated to oligonucleotides, allowing the cargo to be incorporated into a preassembled DNA origami cage (41 × 30 × 21 nm^3^ in size) through 14 addressable ssDNA extensions in its cavity. This technique was shown to release cargo in its unaltered, bioactive state in contrast to existing labile conjugation chemistries, which often interfere with the natural bioactivity of the cargo.

### 5.2. Cellular Delivery of DNA-Enzyme Conjugates

To date, various DNA nanostructures have been successfully transfected into cells in vitro and in vivo [64,65]. Moreover, the transfection rates of intrinsically polar DNA structures could be tuned/enhanced by cationic polymers [60], intercalators [66], lipid membrane formulations [67] and virus capsid proteins [68]. However, so far there exist only a few reported approaches of delivering functional enzymes into cells by taking advantage of DNA nanostructures.

Juul et al. [62] developed a preassembled DNA cage that can encapsulate and release enzymes by temperature-induced conformational changes in the assembly (illustrated in Figure 5e). The cage-like structure was assembled using 12 DNA duplexes that formed the edges of the design. In between the double-stranded domains, there were short ssDNA linkers that made up five cage corners, but the one corner was composed of four 32-nt-long stretches of DNA possessing a sequence that enables hairpin-formation. The hairpin structure was formed only below 22 °C (the annealing temperature of the hairpin-forming sequences), and thus it allowed temperature-controlled switching between the open and closed states. It was demonstrated that the HRP enzyme could enter or exit the cage at 37 °C, but it stayed trapped in the cage when the temperature was lowered to 4 °C (four different states depicted in Figure 4e). In addition, Juul et al. studied if the DNA cage could improve the cellular uptake of HRP enzymes. However, it was observed that the cellular uptake was facilitated only in the presence of Lipofectamine^®^ transfection agent.

Brodin et al. [63] (Figure 5f) showed that cellular delivery of β-galactosidase (β-gal) can be enhanced by coating it with a dense shell of oligonucleotides. The large homotetrameric enzyme was reacted with an excess of a thiol-reactive fluorophore (AF647 maleimide), affording a tetramer with fluorophore modifications (AF4-β-gal). Fluorophores allow the quantification of the protein concentration after modification with oligonucleotides and they provide a handle for monitoring cellular uptake. The formed AF4-β-gal surface was coated with short PEG chain –modified DNA strands (dGGT)_10_ yielding a conjugate ProSNA β-gal with ca. 25 strands of DNA per each tetrameric enzyme (ProSNA β-gal depicted in Figure 4f). The enhancement in the cellular uptake of the complex (~280-fold) resulted from its ability to engage scavenger receptors, which are found on the surfaces of most cells. Importantly, β-gal maintained its catalytic functionality in the transfection process. The method could be generalized to other enzymes as well because the technique does not rely on the properties of the core molecule but, instead, merely on the 3D architecture of the conjugate. 

## 6. Conclusions

Rapid development in the field of structural DNA nanotechnology has recently enabled the controlled self-assembly of complex and functional nano-objects. Unlike most other man-made self-assembled structures, the precise and programmable base-pairing of DNA can be utilized to produce diverse non-symmetric and non-periodic architectures that allow the positioning of functional units with nanometer-level precision. In this review, we have discussed how this compelling property of DNA can be used to control the positioning of enzymes and, furthermore, to affect their capability to catalyze reactions. We hope the discussion and examples presented here provide insight to the relatively young field of DNA-based enzyme reactors and systems, which is currently under heavy development. Some of the hurdles related to the positioning of enzymes and controlling their reactivity have already been solved; however, we expect to see even greater progress in studying how these conjugates, reactors and other systems behave in connection with living organisms.

## Figures and Tables

**Table 1:** Examples of DNA-based enzyme reactors and cascades.

Type	Function	Key Aspects
A glucose oxidase (GOx) – horseradish peroxidase (HRP) cascade on a DNA origami [43].	The enzyme positions on the DNA origami template can be tuned.	The cascade activity is highly dependent on the spacing between the enzymes; the highest activity was found at a 10 nm distance.
A GOx-HRP cascade on a DNA origami that can be rolled into tubular shape [44].	The idea is similar to the above, but here the semi-confined tubular geometry could enable shielding.	The enzymes in the semi-confined geometry show higher enzymatic activity than the free enzyme controls.
A swinging arm between malate dehydrogenase (MDH) and glucose-6-phosphate dehydrogenase (G6pDH) assembled on a double-crossover (DX) DNA tile [45].	The DNA strand acts as a flexible arm that channels the cofactor transfer between the hydrogenases in the complex.	The enzyme activity achieved by the swinging arm is significantly higher than in the case of freely diffusing cofactor.
A tubular DNA origami nanoreactor with GOx-HRP pairs [46].	The nanoreactor is comprised of two units: GOx- and HRP-loaded DNA origamis that can be combined into a complete cascade reactor.	Single origami units and the complete reactor equipped with binding sites show higher activity than the controls without binding sites.
A xylose reductase (XR) – xylitol dehydrogenase (XDR) cascade on a DNA origami [47].	The enzymes are attached to origami via DNA-binding protein adaptors resulting in an artificial enzyme cascade.	The efficiency of the cascade reaction is more dependent on the interenzyme distance than that of the cascade reaction with unimolecular transport between two enzymes.
A three-enzyme pathway assembled by a DNA nanostructure [48].	MDH, oxaloacetate decarboxylase (OAD) and lactate dehydrogenase (LDH) are organized at the corners of the triangular DNA nanostructure, thus forming a three-enzyme cascade.	Activity of the cascade depends more on the geometric patterns of enzymes than the interenzyme spacings.

**Table 2:** Examples of mechanical regulatory DNA-enzyme devices.

Type	Function	Key Aspects
DNA nanotweezers [49,50,51,52] equipped with cascade pairs or with the enzyme and its cofactor.	The tweezers can be opened and closed through a strand-displacement reaction.	The enzyme activity can be controlled by switching the tweezers reversibly.
A tubular DNA origami nanoreactor [53].	The lid of the tube can be opened and closed with the help of lock and key strands.	Flowthrough of the compounds into the confined reaction chamber is controlled by the lid.
A four-arm DNA origami nanoactuator [54].	A distance change in a driver site can be propagated to the mirror site containing binding sites for cargo molecules.	The actuator can be driven using different mechanisms, and it can be used for, e.g., tuning fluorescence behavior of enhanced fluorescent protein (eGFP).
Aptamer-based logical circuit [55].	The autonomous logical circuit controls α-thrombin activity through the convertor, controller and generator modules.	α-thrombin aids blood coagulation, and therefore systems such as this may find intriguing biomedical uses.

**Table 3:** Examples of DNA-based enzyme containers and carriers.

Type	Function	Key Aspects
Hollow DNA origami containers [56,57,58].	These structures could be used in encapsulating molecular cargo.	Examples include successful opening and closing mechanisms for conceivable drug release.
DNA origami half-cages [59] that can be arranged into a closed box.	The closed geometry shields the enzymatic reactions (such as the glucose oxidase (GOx) – horseradish peroxidase (HRP) cascade) against proteases.	Activity of a single enzyme can be enhanced by encapsulating it into the box.
A tubular DNA origami nanocarrier [60].	The carrier acts as a host for luciferase enzymes.	Luminescence of the cargo can be modulated by coating the carrier with cationic polymers.
A box-like DNA origami container [61].	The box facilitates the binding of proteins (such as bovine serum albumin (BSA)) in the cavity of the origami.	Bound proteins can be released by light.
A switchable DNA cage [62].	The cage can trap and release HRP through a conformational change.	The conformational change can be controlled by temperature.
A β-galactosidase (β-gal) protein coated by DNA strands [63].	DNA-coating significantly increases cellular delivery of the enzymes.	The approach is highly modular, and importantly, the enzymes retain their activity in the transfection.

## Abbreviations

The following abbreviations are used in this manuscript:

2D	Two-dimensional
3D	Three-dimensional
β-gal	β-galactosidase
ABTS	2,2′-azino-bis(3-ethylbenzothiazoline-6-sulphonic acid)
AFM	Atomic force microscopy
B-DNA	Double-helical DNA in B-form (geometry attribute)
BSA	Bovine serum albumin
DNA	Deoxyribonucleic acid
dsDNA	double-stranded DNA
DX	Double-crossover
eGFP	Enhanced green fluorescent protein
FRET	Förster resonance energy Transfer
G6pDH	Glucose-6-phosphate dehydrogenase
GOx	Glucose oxidase
HRP	Horseradish peroxidase
LDH	Lactate dehydrogenase
LUC	Lucia luciferase
MDH	Malate dehydrogenase
NAD^+^	Nicotinamide adenine dinucleotide (oxidized)
NADH	Nicotinamide adenine dinucleotide (reduced)
nt	nucleotide
NTV	NeutrAvidin
OAA	Oxaloacetate
OAD	Oxaloacetate decarboxylase
PDMAEMA	Poly(dimethylaminoethyl methacrylate)
PEG	Polyethylene glycol
PMS	Phenazine methosulfate
RNA	Ribonucleic acid
ssDNA	Single-stranded DNA
TMB	3,3′,5,5′-tetramethylbenzidine
XDR	Xylitol dehydrogenase
XR	Xylose reductase
